# mSphere of Influence: Start with an Interesting Biological Phenomenon

**DOI:** 10.1128/mSphere.00299-19

**Published:** 2019-06-19

**Authors:** Teresa O’Meara

**Affiliations:** a Department of Microbiology and Immunology, University of California, San Francisco, San Francisco, California, USA; b Department of Molecular Genetics, University of Toronto, Toronto, Ontario, Canada

**Keywords:** *Candida albicans*, *Saccharomyces cerevisiae*, functional genomics

## Abstract

Teresa O'Meara works in the field of functional genomics of Candida albicans, with a focus on host-pathogen interactions. In this mSphere of Influence article, she reflects on how papers entitled "Systematic Screens of a Candida albicans Homozygous Deletion Library Decouple Morphogenetic Switching and Pathogenicity" by S. M. Noble, S. French, L. A. Kohn, V. Chen, and A. D. Johnson (Nat Genet 42:590–598, 2010, https://doi.org/10.1038/ng.605) and "Exploring Quantitative Yeast Phenomics with Single-Cell Analysis of DNA Damage Foci" by E. B. Styles et al. (Cell Syst 3:264–277.e10, 2016, https://doi.org/10.1016/j.cels.2016.08.008) impacted her research and thinking through pioneering functional genomic screens. These articles show the power of combining defined mutant libraries with screens for interesting phenotypes to understand new biology.

## Abstract (precis)

Teresa O'Meara works in the field of functional genomics of Candida albicans, with a focus on host-pathogen interactions. In this mSphere of Influence article, she reflects on how papers entitled "Systematic Screens of a Candida albicans Homozygous Deletion Library Decouple Morphogenetic Switching and Pathogenicity" by S. M. Noble, S. French, L. A. Kohn, V. Chen, and A.

## COMMENTARY

Functional genomic screens bring together the best of both experimental genetic worlds. They combine forward and reverse genetic approaches to explore compelling phenotypes using defined mutants. Functional genomic screening approaches have proved to be a powerful way to discover gene function, including in the human fungal pathogen Candida albicans, where many of the predicted proteins have no annotated function. The papers “Systematic Screens of a Candida albicans Homozygous Deletion Library Decouple Morphogenetic Switching and Pathogenicity” (1) and “Exploring Quantitative Yeast Phenomics with Single-Cell Analysis of DNA Damage Foci” (2) show the power of functional genomic screens for understanding new biology.

In “Systematic Screens of a Candida albicans Homozygous Deletion Library Decouple Morphogenetic Switching and Pathogenicity,” the authors built the first large-scale homozygous mutant collection for Candida albicans, covering 674 open reading frames, or approximately 11% of the genome. The authors then screened this collection for three phenotypes: growth in defined medium at 37°C, morphogenesis upon incubation in a filamentation-inducing cue, and virulence during systemic infection in a mouse model of candidiasis. The overall goal was to look at genetic determinants of virulence, and by screening *in vivo*, the authors could take an unbiased look at the genes that were required for causing disease. The *in vitro* phenotypes were chosen as proxies for the abilities of the strains to cause disease, and in general, the mutants that had defects in *in vitro* growth or filamentation had a defect in virulence. However, the authors also identified nearly 100 mutants where the virulence defect could not be explained by a defect in morphology or filamentation. This demonstrated that there are additional requirements for C. albicans to cause disease. By examining these mutants in more detail, the authors were able to identify glucosylceramide biosynthesis as a novel requirement for C. albicans during systemic infections.

This paper was one of the first functional genomic screens of Candida albicans, and the created mutant collection was a fantastic resource for the fungal pathogenesis community. This paper was also one of the largest screens for virulence determinants, and the results emphasized that the processes required for virulence cannot necessarily be predicted from *in vitro* phenotypes. Instead, the authors were able to identify new virulence factors based on mutant phenotypes during infection. This paper was inspiring because it started with the most clinically relevant *in vivo* phenotype and let the screening data reveal new aspects of C. albicans pathogenesis that would not necessarily be predicted from *in vitro* tests. This large-scale morphological screening of mutant strains also reinforced the importance of filamentation in virulence, as the genes required for filamentation were, in general, also required for virulence in the host. This work was the inspiration for our genome-scale analysis of morphogenesis.

In “Exploring Quantitative Yeast Phenomics with Single-Cell Analysis of DNA Damage Foci,” the authors expand the definition of a “mutant phenotype” to include modification of protein localization. The authors built a set of fluorescent reporters for Rad52, nuclei, and the cytoplasm in Saccharomyces cerevisiae, thus allowing for quantitative microscopic analysis of foci of DNA damage. When DNA is damaged, the cell assembles a gigadalton-sized protein complex that brings together the proteins required for DNA recombination and repair. One of these proteins is Rad52, which is a protein that facilitates recognition of single-stranded DNA. In a normal cell, there will be one DNA damage focus per cell upon treatment with a DNA-damaging agent, such as phleomycin.

With their different reporters, the authors could then quantify the number and localization of the DNA damage foci, measured by localization of the Rad52-green fluorescent protein (GFP) fusion protein, in different mutant arrays. The arrays included a single mutant array, a single mutant array with phleomycin treatment, a double mutant array where each mutant strain contains an *SGS1* deletion, and a double mutant array where each mutant strain contains a *YKU80* deletion. Together, this encompassed 24,000 mutant populations that were screened by automatic microscopy. Through these screens, the authors identified new modifiers of the yeast DNA damage response by observing changes in Rad52 focus number and localization in the cell. Even in the well-studied model yeast, this approach gave us new information about a conserved process.

This paper was one of the first papers to take advantage of high-content microscopy as a screening tool for functional genomics. This changed the way that we can think about mutant phenotypes, allowing the field to move beyond viability or growth as a quantitative phenotype. The automated and quantitative microscopy pipeline developed in this paper is a phenomenal resource that can be used for many other microscopy-based screens. In addition to being used to examine yeast biology, the tools and pipelines can be applied to many other organisms, especially with the increase in the number of available fluorescent reporter tools. I expect that new genome-scale genetic manipulation techniques for C. albicans will soon allow for this type of detailed phenotypic analysis and drastically change what we can measure in this model eukaryotic pathogen.
